# Antibiotic-Mediated Plasmonic-Mie Resonance for Biosensing Applications on a Novel Silicon Nanopillar Metasurface

**DOI:** 10.1002/admi.202400945

**Published:** 2025-03-31

**Authors:** Jacob Waitkus, JaeWoo Park, Theodore Ndukaife, Sui Yang, Ke Du

**Affiliations:** Chemical and Environmental Engineering, University of California Riverside, Riverside, CA 92521, USA; Material Science and Engineering, School for Engineering of Matter, Transport and Energy Arizona State University, Tempe, AZ85281, USA; Material Science and Engineering, School for Engineering of Matter, Transport and Energy Arizona State University, Tempe, AZ85281, USA; Material Science and Engineering, School for Engineering of Matter, Transport and Energy Arizona State University, Tempe, AZ85281, USA; Chemical and Environmental Engineering, University of California Riverside, Riverside, CA 92521, USA

**Keywords:** antibiotics, antimicrobial resistance (AMR), gold nanoparticles, localized surface plasmon resonance (LSPR), Mie resonances, nanopillar metasurfaces

## Abstract

This study demonstrates a biosensing platform facilitated by localized surface plasmonic resonance (LSPR) on a silicon (Si) nanopillar metasurface mediated by the presence of cephalexin (Cef) antibiotics in solution. The metasurface is designed to exhibit narrow quadrupolar Mie resonances that when coupled with bovine serum albumin-coated (BSA-coated) plasmonic gold nanospheres (BSANS) will produce an appreciable redshift at the peak resonance wavelength, occurring only in the presence of the target antibiotic. To optimize the performance of the Si nanopillars, the finite element method is utilized to fine-tune their diameters, heights, and periodicity, along with improvements to the fabrication techniques, under the BSANS-antibiotic binding assay. The metasurface sensor is directly fabricated via a facile photolithographic process using silicon wafers. Through the detection assay, this device exhibited a significant 22 nm wavelength shift resulting from changes to the local refractive index in the presence of the BSANS-antibiotic coupling. This phenomenon is facilitated through the presence of cephalexin down to 0.3 μg mL^−1^ for the binding between the plasmonic nanoparticles and the metasurface allowing for sensitive and real-time detection.

## 1. Introduction

Overuse and misuse of common antibiotics has promoted antimicrobial resistance (AMR) to become a forefront issue for healthcare professionals.^[1,2]^ Popular medicines such as penicillin are often prescribed as generic remedies when more specialized antibiotics would be appropriate for treatment.^[3,4]^ This fallacy has allowed bacterial mutations to survive and reproduce, causing an increase in new resistances and the number of ineffective treatments. In sepsis patients, rapid and potent medication is required to combat the deadly ailment often caused by bacterial infections.^[5–8]^ The need for identifying AMR in these instances is thus critical. Blood work is commonly performed to identify sepsis in patients, but this detection method is not specific for the identification of AMR.^[9]^ To identify and monitor AMR, current techniques often involve agar dilutions and next generation sequencing (NGS). Agar dilutions are considered a gold standard for this field, cultivating multiple plates of bacteria colonies that have been exposed to varying concentrations of an antibiotic solution.^[10]^ The survival rate of the colonies can be recorded to determine the minimum inhibitory concentration (MIC) necessary to rid the sample of infection for a given species of bacteria. However, this process is time-consuming, requires trained professionals to operate, and is singleplexing. The NGS method allows for complete mapping of the genome from extracted DNA samples of a target bacteria cell, in order to form a library of the genetic determinants.^[11,12]^ This allows for easy identification of mutations in the bacteria sample that could indicate AMR. However, operating and performing NGS takes specialized equipment and larger time constraints to properly establish the genetic database which is not suitable for the timely detection of AMR in sepsis patients. Comparatively, miniaturized biosensing devices may offer real-time detection and simplistic operation to promote widespread use and aid for treating afflictions.

Recent advancements in biosensing have seen the implementation of metasurface-based devices that can improve upon the aforementioned techniques. This is achieved through manipulation of electromagnetic waves by the unique organization of nanoscale structures smaller than the incident light wavelength, resulting in the metasurface.^[13–15]^ Through blocking or absorbing photons, enhancing the intensity of the incoming light, or altering the polarization of the wave, metasurfaces are able to be customized for various bioapplications.^[16,17]^ Popular devices are able to create variable refractive index values or promote high quality factors in order to boost the sensitivity of the surface to changes in their surrounding media, improving detection limits.^[18,19]^ In this work, the electromagnetic waves were manipulated to promote Mie resonance, a form of light scattering that occurs between light waves and metallic or dielectric structures on the scale of the light wavelength.^[20]^ This can result in a multitude of novel effects such as enhanced sensitivity between light and the scattering material with directional scattering for imaging, unique absorbance spectra for biosensing applications, or producing nonlinear and topographical optical systems.^[15,21]^ Mie resonant structures commonly exhibit the capability to support both electric and magnetic resonance, referred to as multipole modes or moments.^[20,22]^ Silicon nanofeatures are often used in this field as their high refractive index and dielectric characteristics within the visible spectrum allows for greater control over the location and intensity of the Mie resonance wavelengths.^[20,23,24]^ Through interactions amongst dielectric silicon and a metallic nanoparticle such as gold, LSPR signals may be promoted.^[25,26]^ This results from exchanges and interactions at sensitive electric quadrupolar sites on the silicon nanopillar, devising a useful sensing platform through coupling metasurfaces with plasmonic systems.^[22,25,26]^ Recent research has seen a multitude of metasurfaces be applied to the biosensing field for the detection of DNA/RNA,^[19]^ protein structures,^[27]^ and antibodies.^[28]^ These miniaturized devices have been developed to manipulate THz waves or optical lasers for identifying viral or bacterial targets based on the surface orientation, geometry, and periodicity of the nanoscale features for the detection of weak or dilute analyte signals.^[19,28]^

In addition, LSPR-based detection can be facilitated through metallic nanoparticles or clusters of these particles, which promotes the collective oscillation of the conductive electron clouds in the nanomaterials. This is done in order to produce hot spot regions where incident photons undergo drastic intensity enhancements.^[29]^ These hot spots form in the spacings between particles as the nanoparticles align their dipoles to work in unison and enhance their electron fields.^[29,30]^ This effect is observed only in close proximity to the particle surface, within tens of nanometers, as the enhancement factor exponentially diminishes with distance from the material.^[30,31]^ Our previous work has demonstrated this effect with LSPR-based detection of viral RNA via gold nanoparticles (AuNP) functionalized with single-stranded DNA (ssDNA).^[32]^ Further, coinage metals are typically employed to achieve LSPR enhancement as their collective oscillations occur within the visible spectrum or NIR region, allowing for more readily detectable changes at the particle surface and for monitoring of chemical reactions.^[33]^

In this work, we demonstrate a novel biosensing device mediated by the presence of cephalexin antibiotics, through the combination of LSPR enhancement and Mie resonance at the surface of a specialized Si nanopillar metasurface. This biosensing material resulted from facile and rapid fabrication techniques to take advantage of Mie scattering in the form of electric quadrupolar resonance signals. Further, the implementation of metallic particles to combine plasmonic detection with the Mie scattering on the nanopillar surface highlights a unique sensing strategy. The use of antibiotics as a means to facilitate these Mie-plasmonic signals is not something previously explored in literature, resulting in a real-time and sensitive biosensor at clinically relevant concentrations for cephalexin identification. The silicon nanopillar metasurface was devised to produce a distinctive reflectance signature utilizing high-order Mie resonance, with a narrow full width at half maximum (FWHM) value below 25 nm. The size parameters and inter-pillar distances of the metasurface were investigated to facilitate the optimal coupling of antibiotic linkers with AuNPs, specifically BSANS, that would preferentially bind to the functionalized nanopillar features. Implementing this LSPR-Mie metasurface technique, an antibiotic-mediated detection assay was developed, enabling a 22 nm wavelength redshift resulting from changes to the local refractive index in the presence of the BSANS-antibiotic coupling with cephalexin at concentrations as low as 0.3 μg mL^−1^.

## 2. Results and Discussion

### 2.1. Nanopillar Metasurface Array Design

**Figure 1.**
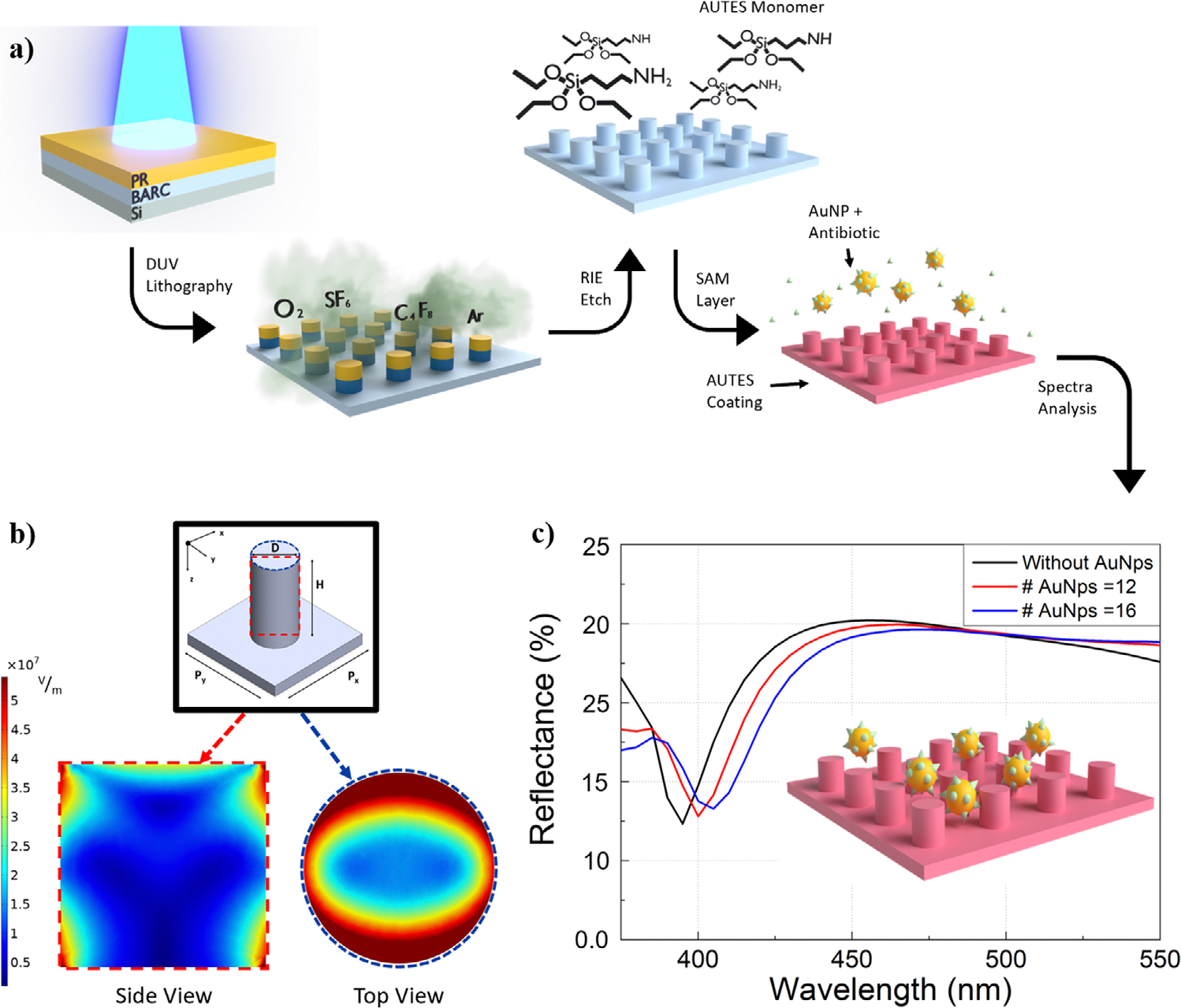
a) Schematic of the fabrication, functionalization, and subsequent testing mechanism for the nanopillar metasurface based on detection of a spectral shift via plasmonic nanoparticle-Mie resonance coupling. b) Design of higher order mode Mie resonance for the Si nanopillar metasurface (D = 180 nm, H = 180 nm, Px = Py = 370 nm). E-field map shows quadrupole distribution along the height of the nanopillar with the field enhancement majorly on the pillar surface, ideal for sensing applications. c) Simulated reflectance spectrum of the metasurface with and without BSANS coupling (Inset: binding event illustration). Simulated even with the small amount of surface binding/coverage of BSANS, the spectral shift is appreciable.

The design principle, fabrication, functionalization, and testing mechanism of our metasurface sensor is depicted in Figure 1a. To boost spectral sensitivity and facilitate on-chip integration, we developed a straightforward Si nanopillar metasurface on a standard Si wafer substrate that manifests high-order quadrupolar Mie resonance, as shown in Figure 1b. This design inherently yields a much narrower resonant peak (~25 nm) compared to conventional dipolar Mie resonances at visible frequencies, offering an excellent platform for spectral shift sensing.^[23,24]^ Moreover, the electric field (E-field) map reveals that the field enhancements of the designed quadrupolar resonance were predominantly concentrated on the surfaces of the nanopillars (top and sides), which further promoted the detection of surface binding events. Thus, the size parameters and inter-pillar spacing detailed in Figure 1b had been meticulously optimized (D = 180 nm, H = 180 nm, Px = Py = 370 nm) to facilitate spectral linewidth and BSANS binding interactions. This optimal binding with BSANS was also confirmed experimentally at different nanopillar parameter sets (Figure S1, Supporting Information). Upon attachment of BSANS to the metasurface, the coupling between the Mie modes of the nanopillars and the LSPR of the gold nanoparticles intensified the local fields, thereby amplifying sensitivity and leading to a substantial spectral redshift. As illustrated in Figure 1c, even with a small number of BSANS bound on the metasurface, the resulting spectral shift was appreciable, enabling precise antibiotic detection.

### 2.2. Fabrication Optimization

**Figure 2.**
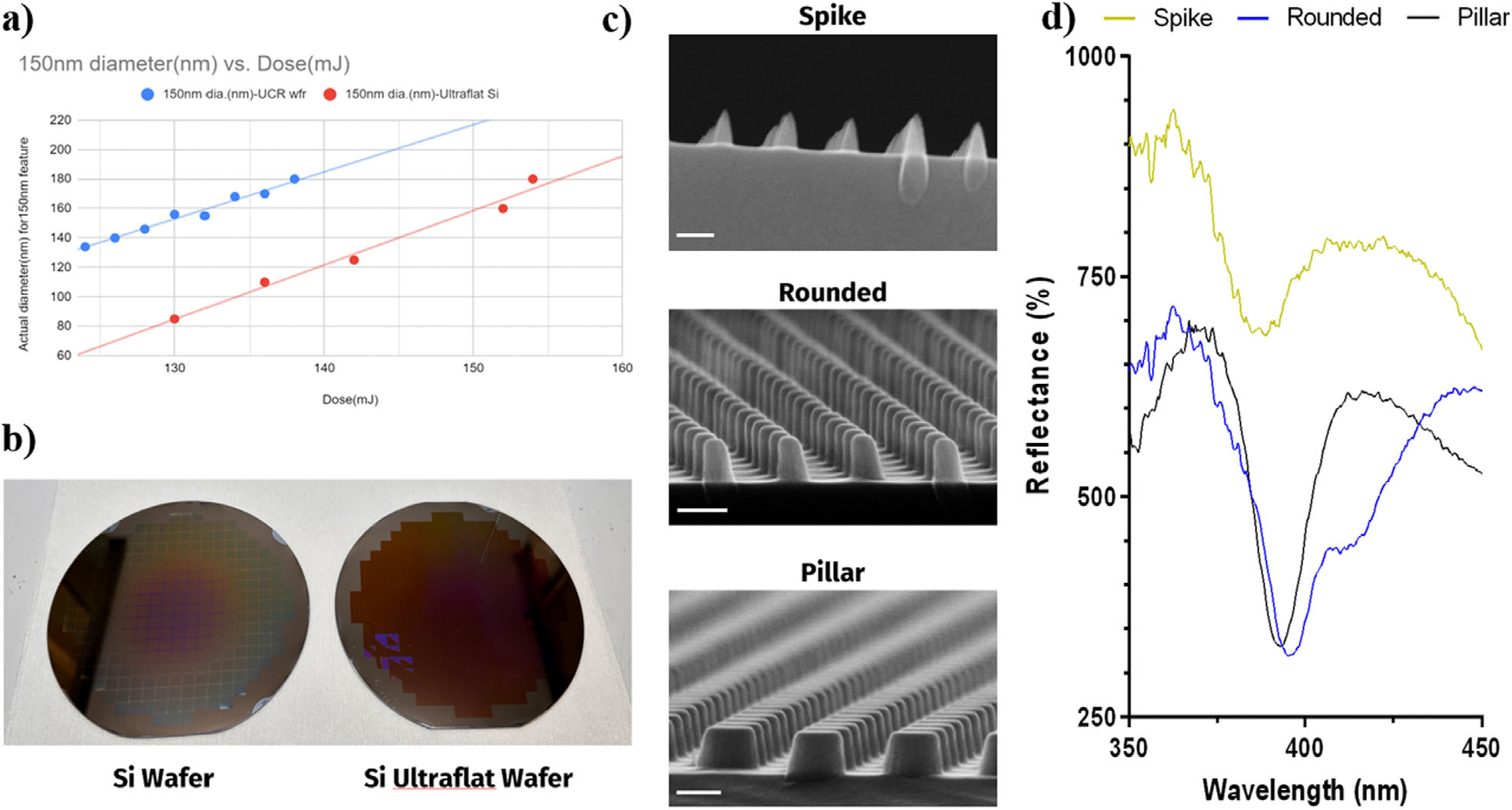
a) Graph for the effect of wafer thickness variation on the DUV dosage required for patterning of specific size PR circles. b) Optical images of wafers after DUV exposure, highlighting the PR variations on high thickness variation wafers. c) SEM images of three diced samples from the edge of the test wafer to the center of the test wafer after etching. (Scale Bars: 200 nm) d) Reflectance spectra for the three adjacent SEM images.

The dimensions of the metasurfaces developed here pushed the resolution limit of common DUV lithography tools. To achieve the desired nanopillar pattern (Diameter: 180 nm, Spacing: 210 nm, Height: 210 nm), the system needed to be optimized for the realization of the uniform photoresist/bottom antireflective coating (PR/BARC)layer across the entirety of the wafer. This specific nanopillar pattern was chosen due to the presence of sensitive reflectance peaks when compared to larger and smaller features (Figure S1, Supporting Information). Additionally, these features showed the greatest repeatability through the lithography and etching process across the wafer surface. This was done through a focus exposure matrix (FEM) study to determine the optimal dosage to achieve the desirable nanopillars. The total thickness variation (TTV) of the silicon wafers proved to be a major contributor to the variations observed in the PR layer when performing the FEM. For an ultraflat wafer, with a TTV below 2 μm, the actual diameter of a 150 nm circle pattern varied greatly from standard silicon test wafers as demonstrated by Figure 2a where the dosage exposure in mJ was compared to PR diameter of the nanopillars. The importance of the wafer thickness variations can be observed optically in Figure 2b as the variations in PR circle diameters and thickness produced a color change across the surface of the wafer. This was further apparent in the SEM images and accompanying spectra, highlighting the widened or suppressed reflectance peaks produced in Figure 2c,d. For samples from different locations on the wafer where improperly exposed PR fails to protect the underlying silicon partway through the etch process, we observed nonideal nanopillar structures with weaker or broadened Mie resonance signals that were undesirable for biosensing. The thinner PR layers produced spike-like structures after etching which resulted in lower intensity peaks in the novel reflectance spectrum, more akin to that of the planar silicon substrate. The diminished effect was apparent in comparison to the nanopillar structures, where a sharper reflectance valley was observed in the visible region for the optimized lithography process.

**Figure 3.**
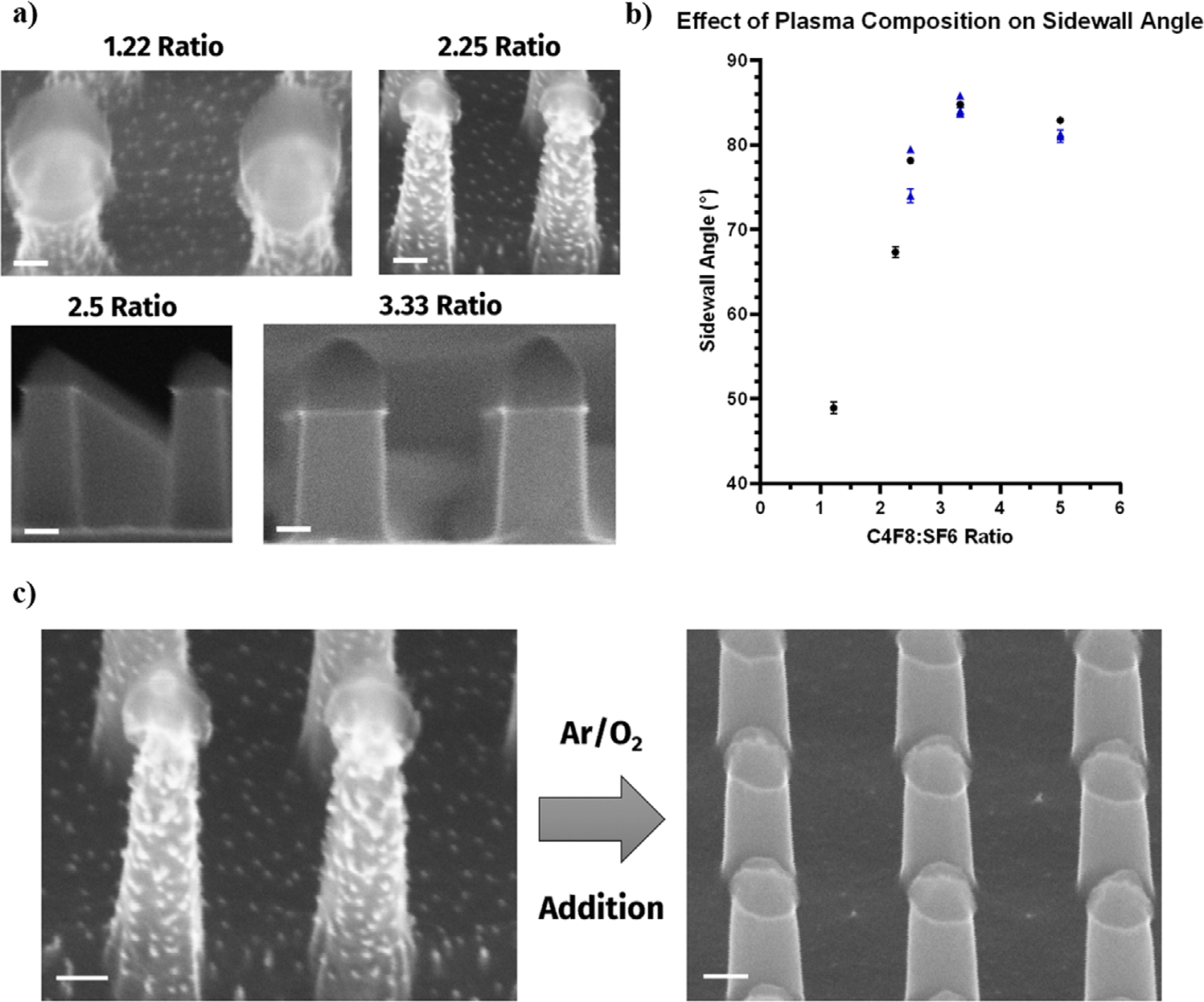
a) SEM images following plasma etching of the Si nanopillar substrate under differing ratios of C_4_F_8_:SF_6_ gas flow rates. (Scale Bars: 100 nm) b) Graph for the effect of C_4_F_8_:SF_6_ ratio on the sidewall angle of the Si nanopillars. c) SEM images highlighting the surface cleanliness following Ar/O_2_ additions to the etch plasma. (Scale Bars: 100 nm).

To further ensure the optimal metasurface fabrication, the sidewall angle of the nanopillar was further investigated. Improving the sidewall angle helped to suppress size inhomogeneity and promote narrow reflectance peaks, thereby improving the sensitivity of the detection system. In the etch plasma, the ratio of C_4_F_8_:SF_6_ gases were controlled between 1.22 to 5.00 as the sidewall angle was recorded by SEM images and plotted in Figure 3a,b. It was observed that above a ratio of 3.33 the sidewall angle plateaued ~84° and slowly decreased as the C_4_F_8_ ratio was increased. As the concentration of carbon in the plasma increased with respect to fluorine radicals, a greater passivation of the silicon surface occurs. This helped to promote a more anisotropic characteristic to the etch which limited undercutting of the PR/BARC layer. As the etch proceeded deeper into the silicon, the sidewalls undergo polymerization from the CF_x_ radicals in the plasma leading to the observed increase in sidewall angle. Under the RF bias, the ions in the plasma were accelerated to the surface creating a physical etch that will ignore the sidewall polymers and preferentially etch the underlying substrate, thereby further improving the sidewall angle and providing height to the nanoscale structures. This effect was also controlled by the pressure value which alters the mean free path of the ion and affects the physical or chemical etch characteristic. With the implementation of the physical etch, the addition of O_2_ gas aided in removal of the sidewall polymers while the Ar was crucial for diluting the F ions in the plasma and carrying away etched species. The clean surface observed in Figure 3c resulted in the desirable silicon nanopillars capable of producing the reflectance spectra unique to this metasurface.

### 2.3. Antibiotic-Based Detection

**Figure 4.**
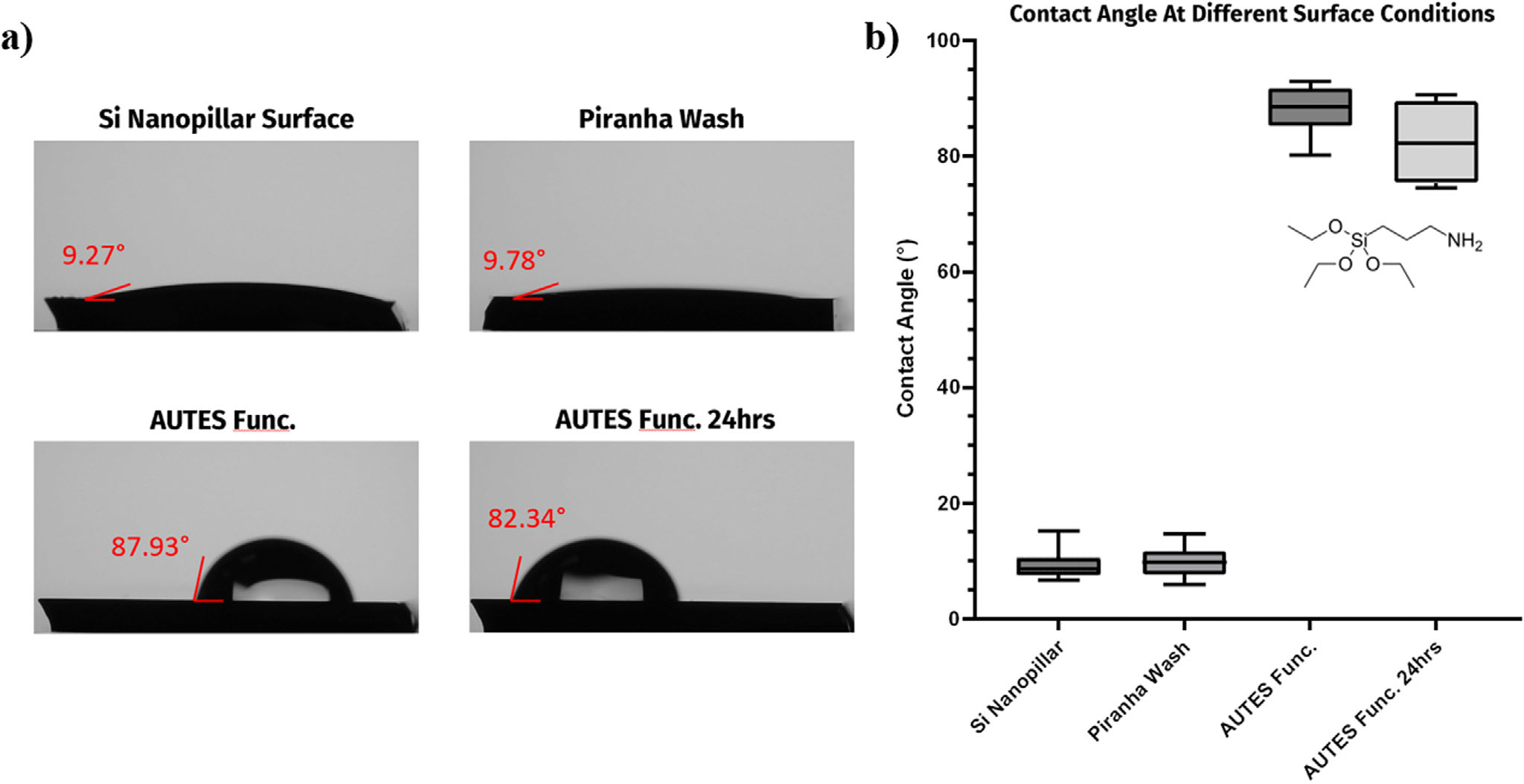
a) Contact angle images of the Si nanopillar surface after various process steps. b) Box and whisker plots for the average contact angle following the steps of Si nanopillar preparation and functionalization (One-way ANOVA MATLAB R2022b, n = 9, P = 1.14e-14).

To best promote the LSPR-Mie effect between the BSANS and silicon nanopillars, a coupling strategy was implemented. This consisted of an 11-aminoundecyltriethoxysilane (AUTES) monomer SAM layer functionalization of the nanopillars with a glutaraldehyde (GLU) spacer chain present on the BSANS. The binding of these components was mediated by the presence of the cephalexin antibiotic as a linker. A multitude of different methodologies were tested to optimize the AUTES monomer layer to limit cross-linkage and multilayer formation between monomer constituents (Figure S2, Supporting Information).^[34,35]^ The crucial factor in ideal formation of the monolayer was limiting the presence of water molecules at the surface. H_2_O molecules played an important role in starting the hydrolysis reaction of the siloxane groups on the AUTES chain but may lead to uncontrolled cross-linking of the monomer units if present in large quantities.^[36]^ The resulting ethoxy moieties in the monomer preferentially bound to the hydroxyl groups of the native silicon oxide layer resulting from the piranha bath described in the experimental methods. Mixing the monomer solution with a toluene solvent helped to ensure controlled monolayer formation in combination with a post-curing step that aided in alignment. The presence of the SAM layer was confirmed via contact angle measurements preceding and following each process step. With the amino terminal group coating the surface, a detectable increase in the hydrophobicity of the nanopillar surface was observed and reported in Figure 4a. The AUTES coating was recorded again after 24 h to verify the stability of the monomer when exposed to the moisture in the atmosphere, which has been shown to cause degradation of the AUTES monolayer over time.^[36]^ The contact angle was shown to increase from ≈9° to over 80° via the addition of the SAM layer to the metasurface confirming the proper alignment of the terminal amino groups from the AUTES monomer on the surface of the nanopillars (Figure 4b). Following exposure to the atmosphere at room temperature for 24 h, the contact angle of the surface still demonstrated statistically similar hydrophobicity, confirming the stable orientation of the monolayer. This may be attributed to the choice of monomer constituent, as AUTES units were chosen over (3-Aminopropyl)triethoxysilane (APTES) for its longer carbon chain. As a result, greater shielding and protection to the bound silanol groups on the silicon oxide occurred, providing higher stability and improved organization in the initial formation of the structures.^[36,37]^

**Figure 5.**
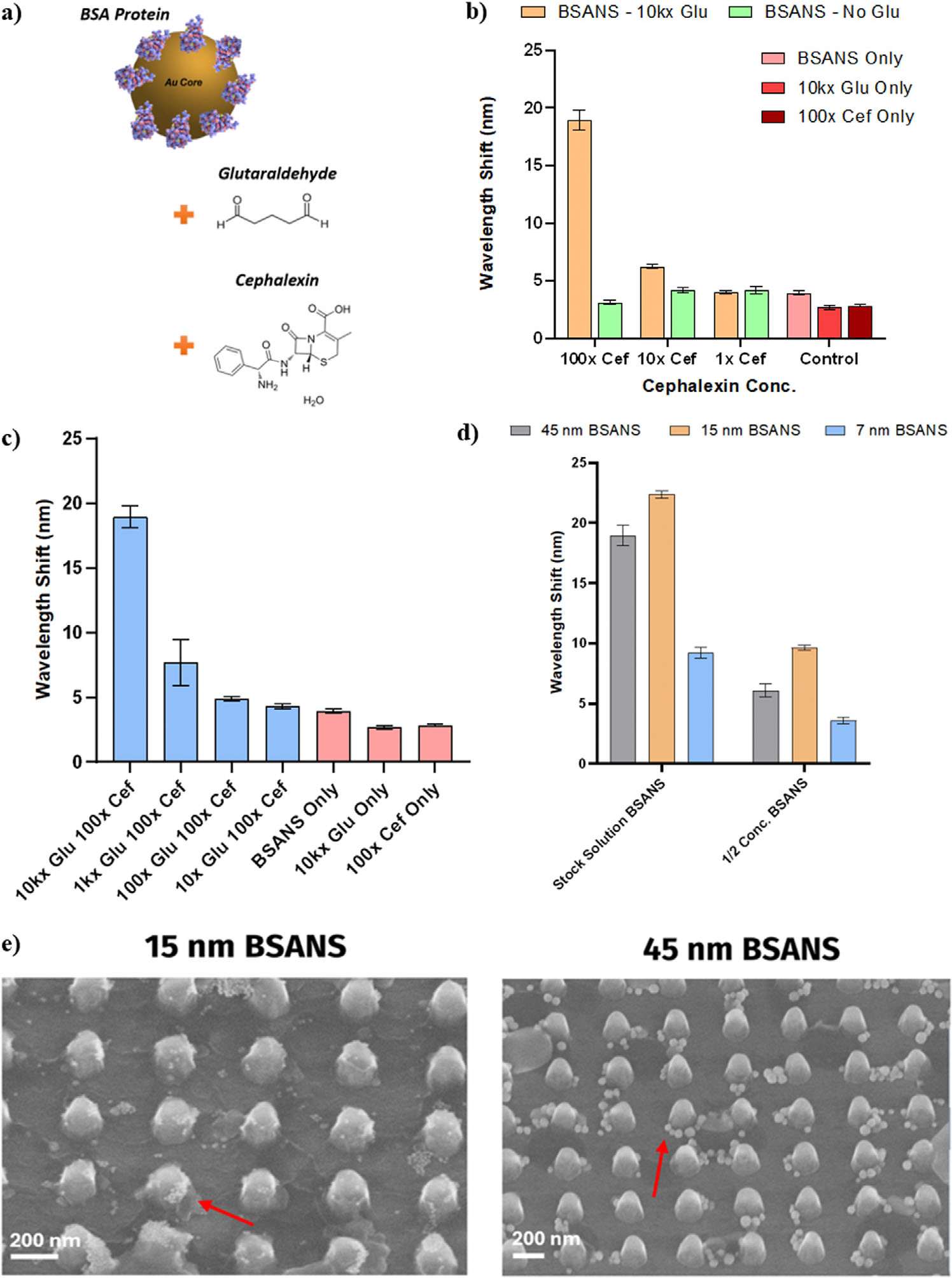
a) Model of the BSANS linker coupling for binding on the functionalized Si nanopillars. b) Variations to the concentration of Cef in the presence and absence of GLU spacers to show a LoD of 0.3 μg mL^−1^ at 10kx GLU and 10x Cef (One-way ANOVA MATLAB R2022b, n = 3, P = 2.199e-17). c) Study on the effects of altering the GLU ratios for the maximum BSANS capture across the nanopillar features (One-way ANOVA MATLAB R2022b, n = 3, P = 1.162e-14). d) Effect of Au core diameter on the redshift magnitude detected under different nanoparticle concentrations (One-way ANOVA MATLAB R2022b, n = 3, P = 1.382e-10). e) SEM images for the 15 nm and 45 nm BSANS particles, showing the coverage of the pillars under identical assay conditions. (Scale Bars: 200 nm).

The capture of the BSANS by the AUTES amino groups was promoted by the formation of a linker chain, consisting of the GLU and Cef antibiotic as demonstrated in Figure5a. The formyl groups on opposing ends of the GLU spacer were bound to the terminal amino group of lysine chains found within the BSA binding domains, and the amino group located within the cephalexin molecule.^[38,39]^ This resulted in the formation of the BSANS-GLU-Cef complex to later be detected across the metasurface. Without the addition of the GLU molecule, the Cef antibiotics were unable to effectively bind with the AUTES monolayer as they became completely enveloped in the binding domains of the BSA proteins.^[40,41]^ As such, the GLU was a crucial element of the assay, acting as a spacer molecule to increase the sensitivity of the system. This aforementioned construct was formed in solution, prior to exposure to the sensing surface. Figure 5b,c graphs the resulting redshift in reflectance peak wavelength under differing ratios for the Cef and GLU molecules respectively. In these figures, the red bars are representative of control samples where only the individual components are added to the metasurface. The optimal condition was observed to be at 10000 times greater GLU molecules in solution than the amount of BSA binding sites and 100 times greater cephalexin compounds available when compared to the number of BSA proteins on the gold nanoparticle, where the wavelength shift detected was ≈19 nm. The oversaturation of the linkers allowed for an increased functionalization of the BSANS binding locations to improve their capture rate on the silicon features. The capture efficiency in these instances was not directly measured but were estimated to be between 10% and 15% based on comparisons to human albumin in the bloodstream, which is chemically similar to that of the BSA protein.^[42,43]^ These ratios were utilized based on clinical levels of cephalexin often found in patients, where their bloodstream may contain between 7.7 and 18 μg mL^−1^ depending on the dosage of the medication.^[44,45]^ Further, from Figure 5b, it was observed that 10x Cef solutions were able to be statistically distinguished from control samples as well as experiments where the BSANS particles are coupled with the Cef in the absence of the GLU spacer. In the case of the control sample where only 100x cephalexin (3 μg mL^−1^) was added to the nanopillars, a signal less than 4 nm was observed. Positive identification of samples with 10x Cef in solution equates to ≈0.3 μg mL^−1^ cephalexin concentration, which is the limit of detection for this work and below clinically relevant levels of cephalexin by a factor of 10 to 100 times. This instance resulted in a redshift of ≈5.5 nm across the nanopillar metasurface, while 1x Cef experiments produced less than the 4 nm observed for the pure cephalexin addition without any nanoparticles present.

Further, as the presence of the GLU decreased in solution (Figure 5c), the appreciable wavelength shift decreased to a minimum redshift ≈3 nm, at constant concentrations of cephalexin. This result aligns with the protein envelopment concept previously mentioned, as without a spacer the gold core would struggle to combine with the silicon metasurface since the BSA protein will completely surrounded the antibiotic linker via transformational structure changes, only producing occasional non-specific binding resulting in the small-scale shifts detected here.^[40,41]^ Additionally, the optimum diameter for the gold core was investigated at different linker component concentrations in Figure 5d to maximize the LSPR effect. For 15 nm BSANS particles, a stronger redshift was observed at 400 nm for the reflectance spectrum when compared to the 7 and 45 nm particles. This observation was attributed to the binding locations of the 15 nm BSANS on the nanopillars when compared to that of the 45 nm BSANS. As previously discussed, the electric quadrupolar activity of the metasurface primarily occurs along the sidewall and top edge of the nanopillar features. Through the SEM images in Figure 5e, the 45 nm particles fail to consistently cover the nanopillars effectively and prefer to bind at the point where the substrate and the nanofeatures meet. In contrast, the 15 nm particles demonstrate not only greater aggregation amongst themselves, but exhibit the greatest coverage of the nanopillars, resulting in stronger interactions with the metasurface and therefore larger redshifting.

Furthermore, the functionalization of the gold nanoparticles with BSA proteins allows for the facile capture of the cephalexin/GLU linker chain, in addition to preparing the assay for future sensing in realistic environments such as milk, blood, and urine. These complex solutions are often protein-rich environments and are comprised of numerous non-target molecules that could lead to a lower sensitivity in identifying cephalexin antibiotics. Previous literature has reported the role of BSA in blocking the non-specific binding of these background components to the biosensor.^[46,47]^ As such, incorporating BSA directly on the surface of the gold nanoparticles helps in blocking undesirable proteins and antibodies from functionalizing with the gold core which improves the specificity of the assay for the target antibiotic of interest.^[48]^ The solubility of the nanoparticles also improves in addition to decreasing the immunogenicity as a direct result of the BSA-coating on the gold core.^[48,49]^

In comparison to alternative biosensors, the detection limit for cephalexin is less sensitive in this work but still within relevant detection ranges as mentioned previously for cephalexin in the bloodstream. The advantage of this device over different biosensors, such as electrochemical and voltammetric devices, is the facile and rapid detection strategy outline herein.^[50,51]^ The limited operating equipment necessary in this instance is advantageous in forming a more portable biosensor with reasonable capabilities for point-of-care detection. The simple two-step fabrication procedure developed through this work to produce highly sensitive Mie resonant peaks is a particular advantage of this device for real-time sensing based on reflectance measurements. Alternative nanofeatures may incorporate a variety of fabrication techniques such as nanoimprint lithography, electron beam lithography, and self-assembly.^[32,50,52,53]^ These each offer their own collection of advantages and disadvantages when compared to the steps used in this work. Self-assembly and nanoimprint offer techniques for larger scale reproducibility and rapid formation of nanoscale features at the cost of consistency or spatial control in the case of self-assembly.^[32,53]^ In comparison, electron-beam lithography and the DUV patterning used herein are more time consuming but provide greater resolution and control of the feature spacing and organization.^[52]^ Further, the shortcomings of electron-beam lithography is the scalability, limiting the ability to cover larger areas of a substrate which the DUV strategy used in this publication may easily achieve.

## 3. Conclusion

We have designed, fabricated and tested a silicon nanopillar metasurface capable of the sensitive detection of AuNPs in the presence of a target antibiotic through plasmonic-Mie resonance interactions. Incorporating antibiotics to facilitate these effects has not been meticulously explored in literature. The biosensor produced herein demonstrated the capability to sense cephalexin in solution at concentrations of 0.3 μg mL^−1^ as a result of changes to the local refractive index. The precise detection of cephalexin was achieved by delicate control over the high-order Mie spectral features to produce the novel reflectance spectrum with a narrow FWHM value that when combined with LSPR coupling would promote large-scale local electromagnetic field enhancements and refractive index changes. Through a facile photolithography production process, the geometry of the silicon nanopillars was optimized to best promote the simulated effect. Control over the sidewall angle of the pillars and limiting the undercutting of the PR/BARC layer proved crucial during the etching for production of the metasurface structures. From combination of the metasurface with LSPR enhancement, the capture of BSANS on the surface provided a mechanism for detection of the presence of cephalexin in solution. Implementation of the GLU molecule as a spacer was to improve the sensitivity of the testing assay. Without the presence of the GLU molecule, the protein binding domains envelop the cephalexin antibiotic. This encompassing creates steric hinderance that limits the ability for the antibiotic binding sites to couple with the AUTES amino group on the silicon nanopillars. Further, the formation of multimeric gold clusters and greater nanopillar coverage via optimizations to the BSANS size helped overcome the weaker expected LSPR property of the smaller 15 nm gold cores through stronger interactions with the nanopillar substrate. The realization of this device demonstrates the advantage of real-time and sensitive detection strategies capable for future multiplexing detection.

Future investigations into the optimal nanopillar parameters for realization of unique Mie resonance spectral signatures in different wavelength regions propagates the potential applications of this system to identify various target analytes. Additional work on this aspect would allow for the detection of AMR in the form of enzymatic activity or genetic mutations by varying the linker chain and surface functionalization of the silicon to the appropriate capture materials.^[54,55]^ Through varying nanopillar patterns on a singular wafer, the potential to identify multiple AMR pathways in real-time may be achieved with delicate control over nanoparticle and metasurface design. Additionally, coupling the device with popular techniques such as RPA amplification for MRSA gene detection may help to increase the portability of the device.^[56]^ The testing of this sensor within complex media is also of particular interest as it expands the true applicability of the system to identify and incorporate cephalexin antibiotics as the mediation component with relevant sensitivity and specificity. Through the development of this assay, we reported a pathway to promote plasmonic-Mie resonance via antibiotic capture. The use of the BSA protein as a coating provides a basis for further testing in more complex and realistic environments, such as spiked serums, where the background constituents may interfere with the cephalexin binding process. With the addition of the BSA coating, a greater specificity for the target antibiotic may be achieved as the BSA would prevent non-specific binding across the surface of the gold core, limiting interactions with the non-specific proteins and antibodies.

## 4. Experimental Section

### Nanopillar Fabrication:

For the realization of the nanopillar metasurface detection assay, a facile fabrication process was developed requiring only nanolithography and dry etch processing, followed by the functionalization of the metasurface for integration with the LSPR-based detection assay. The patterning was performed using a deep-UV (DUV) photolithography tool (ASML 5500 stepper) with an exposure area confined to the overlap of a 31 mm diameter circle and a 22 mm × 27 mm rectangle. The mask used herein contained nine various nanopillar patterns on a 6″ quartz wafer (6250E grade; Didgitat-Toppan) allowing for multiple different dimensions to be patterned with ease. The metasurface lithography was achieved via exposure of a 248 nm KrF laser from the DUV tool onto a UVN30 negative photoresist (PR) and a DUV-42P-6 bottom antireflective coating (BARC) that was spun onto a 4″/100 mm ultraflat silicon wafer (Ultrasil) at a thickness of 500 and 80 nm respectively.^[57]^ The unexposed PR layer was removed via an AZ300MIF developer wash. To remove the BARC layer from the unexposed regions, an O_2_ plasma etch was performed in an FL-ICP (Plasma-Therm 770 SLR) tool under 20 sccm of O_2_ at 10 mTorr with a 100 W RF bias for 1 min.^[58]^

The nanopillar formation was finalized by a reactive ion etching (RIE) process to produce the desirable nanopillar height and sidewall conditions. Prior to the etch, the patterned wafers were cleaved by diamond-tipped scribe pens into ≈2 cm^2^ samples and cleaned under an air jet. This was done to gently remove debris that resulted from the cleavage without altering the remaining PR/BARC pattern. The cleaved samples were set on a 4″ silicon carrier wafer and inserted into an atomic layer etching (ALE) tool (PlasmaPro 100 ALE System) that was used in RIE mode. The plasma was comprised of C_4_F_8_, Ar, SF_6_, and O_2_ gases at 50, 30, 15, and 2 sccm respectively. These gases were ignited and controlled by an RF bias at 50 W and ICP power set at 1200 W under 10 mTorr of pressure. This process was run for 2 mins on the empty ALE chamber prior to etching the samples to condition the chamber for production. The etch time was adjusted depending on nanopillar height, where a 51 s was used to produce 210 nm deep nanopillars. To remove the remaining PR/BARC layers, an O_2_/Ar strip was performed in the same chamber with 50 sccm of O_2_, 10 sccm of Ar, at 20 mTorr pressure, a bias of 75 W, and an ICP of 2000 W for 5 mins. Between the etch and the strip step, the samples were removed, and the strip recipe was run to ensure the chamber was properly conditioned to limit any further silicon etching by remaining etchants from the previous step. This allowed for the final realization of ideal nanopillar metasurface structures.

### Testing Apparatus:

Reflectance spectrums were recorded via an Ocean Insight Flame Miniature Spectrometer (Figure S3, Supporting Information). A tungsten halogen lamp (HL-2000 Ocean Insight) was directed to the surface through a collection of fiber optic cables. The cable bundle consisted of six emission fiber optics surrounding the detector probe (QR400–7-SR-BX EOS-A695605 OceanInsight). Silicon nanopillar samples were tested using a Spectralon (Labsphere) background surface standard, which is reflective over 99% for the visible wavelength region. Contact angle measurements were performed using a contact angle goniometer from Ossila. A Canny edge detection algorithm was used to record the contact angle of 5 μL water droplets on the surface of the metasurface. Nanopillar samples were air jet dried to preserve the surface condition.

### Modeling:

To characterize the silicon metasurface performance, 3D electromagnetic simulations using finite element methods were performed within COSMOL Multiphysics. This was done to determine the optimal periodicity, diameters, and nanopillar height to achieve the metasurface reflectance spectrum on the polarization-insensitive metasurface without sacrificing the resonance linewidth/FWHM. For the COSMOL simulations, port boundary conditions were applied along the direction of propagation of incident radiation with periodic boundary conditions along the x and y axes. Perfectly matched layer absorbing boundary conditions were also applied in the z direction. Refractive index changes were also simulated in the presence of bound 45 nm gold nanoparticles to observe the resulting redshift across the complete visible spectrum when compared to the experimental spectrum of the metasurface and a planar silicon sample.

### Antibiotic Assay Design:

Toward the combination of LSPR effects with the Mie resonance quadrupoles, an assay was developed incorporating antibiotics as the linker system between BSANS and the silicon nanopillars. Three sizes of BSANS were acquired from Luna Nanotech at stock concentrations of 4.9 nm for the 45 nm particles, 0.136 μm for the 15 nm particles, and 1.56 μm for the 7 nm diameter BSANS. All three samples were stored at 4 °C where they were stable for six months. A GLU molecule was implemented as a spacer between the BSA coating and the cephalexin antibiotic. The GLU was suspended in aqueous media at 2.5% (Electron Microscopy Sciences) and stored at 4 °C for long-term stability over multiple months. The cephalexin was purchased from Thermo Fisher Scientific as a monohydrate powder and stored at 4 °C as well which demonstrates stability over 90 days. For the self-assembled monolayer (SAM) on the nanopillars, an AUTES monomer was purchased from Gelest, Inc.

The silicon nanopillar surface was functionalized with a SAM layer following a pre-treatment procedure. The cleaved samples were sonicated in acetone and IPA separately for 5 min before being dried via air jet. The samples were then submerged in a piranha solution for 30 min at room temperature. This bath was prepared at a 3:1 ratio of H_2_SO_4_:H_2_O_2_ by volume. Immediately following the piranha solution, the samples were washed in copious amounts of DI water and dried under N_2_ gas. Here, the piranha washing was used to remove any organic contaminants and to form a uniform and thin silicon oxide layer which is preferable for the capture of the AUTES monomer. For functionalization of the silicon with the monomer, the samples were washed in toluene three times to limit water presence on the surface. The AUTES monomer was dissolved in toluene to 2% at 90 °C for 2 h. The ideal solution condition was tested and shown in Figure S2 (Supporting Information). These samples were then washed in toluene three times again to remove any unbound molecules from the surface. The final devices were baked at 110 °C for 30 min to ensure the monomer alignment was ideal and to remove any remaining moisture. Stability of the monolayer was demonstrated via contact angle measurements over a 24-h period where minimal degradation in wettability and hydrophobicity was observed.

The BSANS linker chain was formed by first mixing 20 μL of stock BSANS with 200 μL of GLU (5.3 mg) for the 7 nm and 15 nm particles, while 20 μL of the 45 nm particles were mixed with 20 μL of GLU (.53 mg). This was done to ensure that the ratio of GLU molecules to the number of BSA lysine chains was consistent at 10,000x. The solution was mixed for 5 min before introducing the cephalexin antibiotic, followed by another 30 min of mixing at room temperature. The antibiotic was dissolved in PBS 1x (pH 7.4) to a final concentration of ≈300 μg mL^−1^ for the 7 and 15 nm particles, and ≈3 μg mL^−1^ for the 45 nm particles. Finally, the device was washed in PBS 1x three times, and air jet dried to remove any weakly associated or unbound nanoparticle chains before spectral testing. This allowed for a pre-loading of the antibiotic component prior to combination with the nanopillars.

### Statistical Analysis:

For the preparation of the figures, minimal preprocessing of the represented data was performed. Across all figure sets, no outliers were excluded in the error and standard deviation calculations. For each figure, the unique box plots, column peak values, and spectrum signatures are representative of the mean value across the metasurface sensor. In each data set, three data points were recorded (n = 3) across three separate locations on the nanopillar metasurface, then averaged to give a proper representation of the state of the device for statistical analysis. For the contact angle calculations, nine data points were recorded (n = 9) across the biosensor. The error bars are representative of the standard deviation (SD) of the multiple data points used in the calculation of the mean value. This was to ensure that any variations and differences through experimentation were accounted for that weren’t necessarily represented by the average value. To determine the statistical significance of various data sets, MATLAB 2022b was used to perform one-way ANOVA testing. This was process was utilized for the contact angle measurements prior to and directly after the formation of the AUTES monolayer on the nanopillars. Additionally, one-way ANOVA testing was conducted to analyze the detection limit of the sensor based on cephalexin concentration, glutaraldehyde concentration, and the diameter of the gold nanoparticle. These experiments were compared to control samples of the individual components of the assay when relevant. To confirm statistical differences, a P value below 0.05 was utilized. The one-way ANOVA test assumes that the samples are normally distributed and independent of one another, which is within reason for this assay.

For the comparison of the metasurface contact angle measurements, a P value of 1.14e-14 was observed. Through the box plot, comparison figures, and analysis table in Figure S4a,b and Table S1a (Supporting Information), it was demonstrated that the contact angle for the nanopillar surface following the AUTES functionalization was statistically different from the nanopillar surface prior to the monolayer forming. The analysis performed on the cephalexin concentrations in Figure 5b produced a P value of 2.199e-17. Additionally, this analysis was incorporated in determining the limit of detection for the device where the 10kx Glu 10x Cef data set was shown to be significantly different from the three control samples (Figure S4c,d; Table S1b, Supporting Information). This verified that the sample with 10 kx Glu 1x Cef was not able to produce significant redshifting above the control data. Further statistical testing on Figure 5c for the effect of glutaraldehyde concentrations was performed and demonstrated a low P value of 1.162e-14 (Table S1c, Supporting Information). These results show that below 1kx Glu compared to BSANS particles, the resulting plasmonic effects are not statistically significant compared to the addition of BSANS by themselves (Figure S4e,f, Supporting Information). Final ANOVA tests were done to analyze the difference between gold core variations, showing a P value of 1.382e-10 (Figure S4g,h; Table S1d, Supporting Information). The significance of switching the nanoparticle core diameter to 15 nm from 45 nm highlighted differences in the redshifting that would indicate greater plasmonic effects.

## Supplementary Material

Supplement information

Supporting Information is available from the Wiley Online Library or from the author.

## Data Availability

The data that support the findings of this study are available from the corresponding author upon reasonable request.
