# Supplementary material for: Antibiotic-Mediated Plasmonic-Mie Resonance for Biosensing Applications on a Novel Silicon Nanopillar Metasurface
Source: Adv Mater Interfaces. Author manuscript; Available in PMC 2025 Aug 20. (PMC12364006; doi:10.1002/admi.202400945)
Supplement: Supplement information [file NIHMS2092947-supplement-Supplement_information.pdf]

Supporting Information

Antibiotic-Mediated Plasmonic Resonance on a Novel Nanopillar Metasurface Array

Jacob Waitkus, [A] *JaeWoo Park,* [B] *Theodore Ndukaife,* [B] *Sui Yang,* [B] *Ke Du* [A,] *

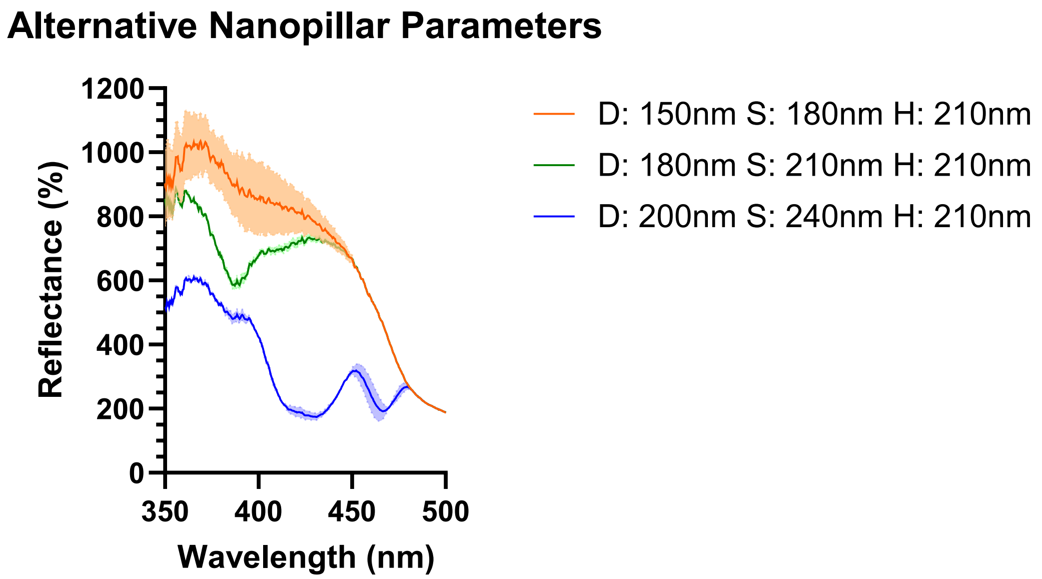

Figure S1. Experimental UV-Vis reflectance spectra of three nanopillar parameters set with differing nanopillar heights, periods, and diameters to highlight the sensitive peaks observed in 180 nm diameter pillars used in this work.

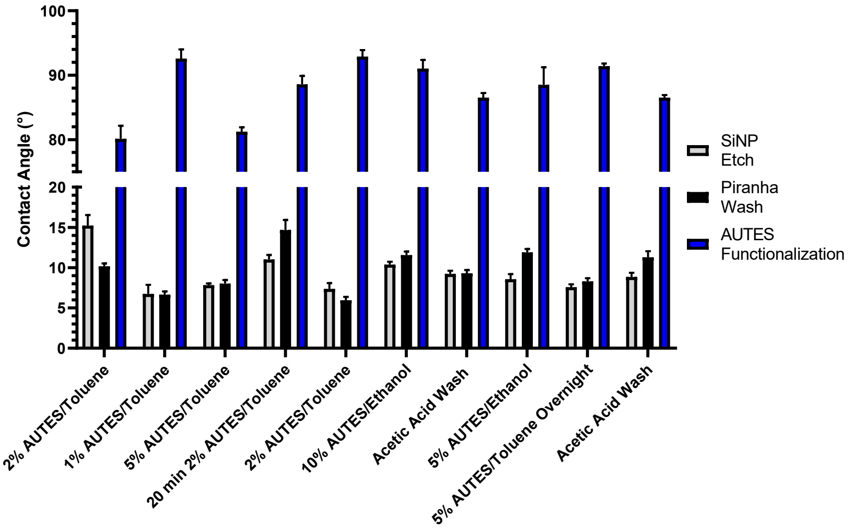

Figure S2. Bar chart for different AUTES functionalization solutions. Solutions of toluene and ethanol were tested to find the optimal alignment and SAM layer formation.

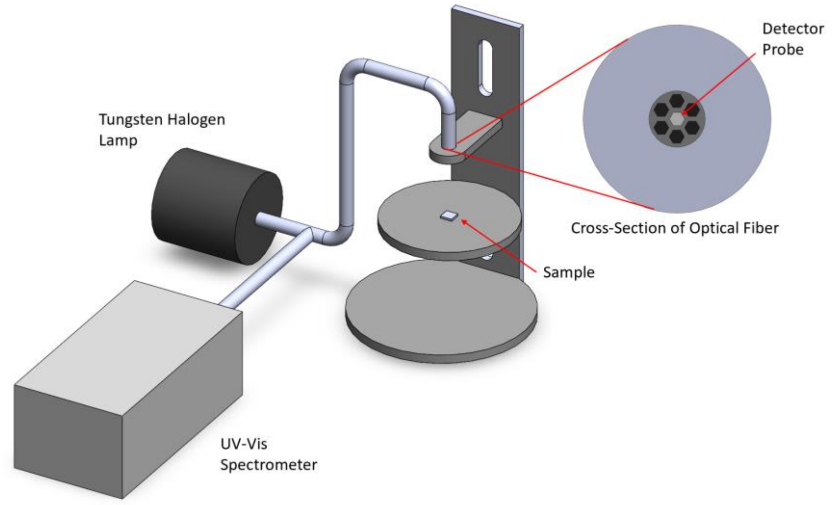

Figure S3. 3D model for the reflectance spectroscopy measurements containing the tungsten halogen lamp light source, the UV-Vis spectrometer, and the Y-branched fiber optic coupling. The cross section of the optical fiber is shown in the top right consisting of six emitter probes surrounding a single detector probe.

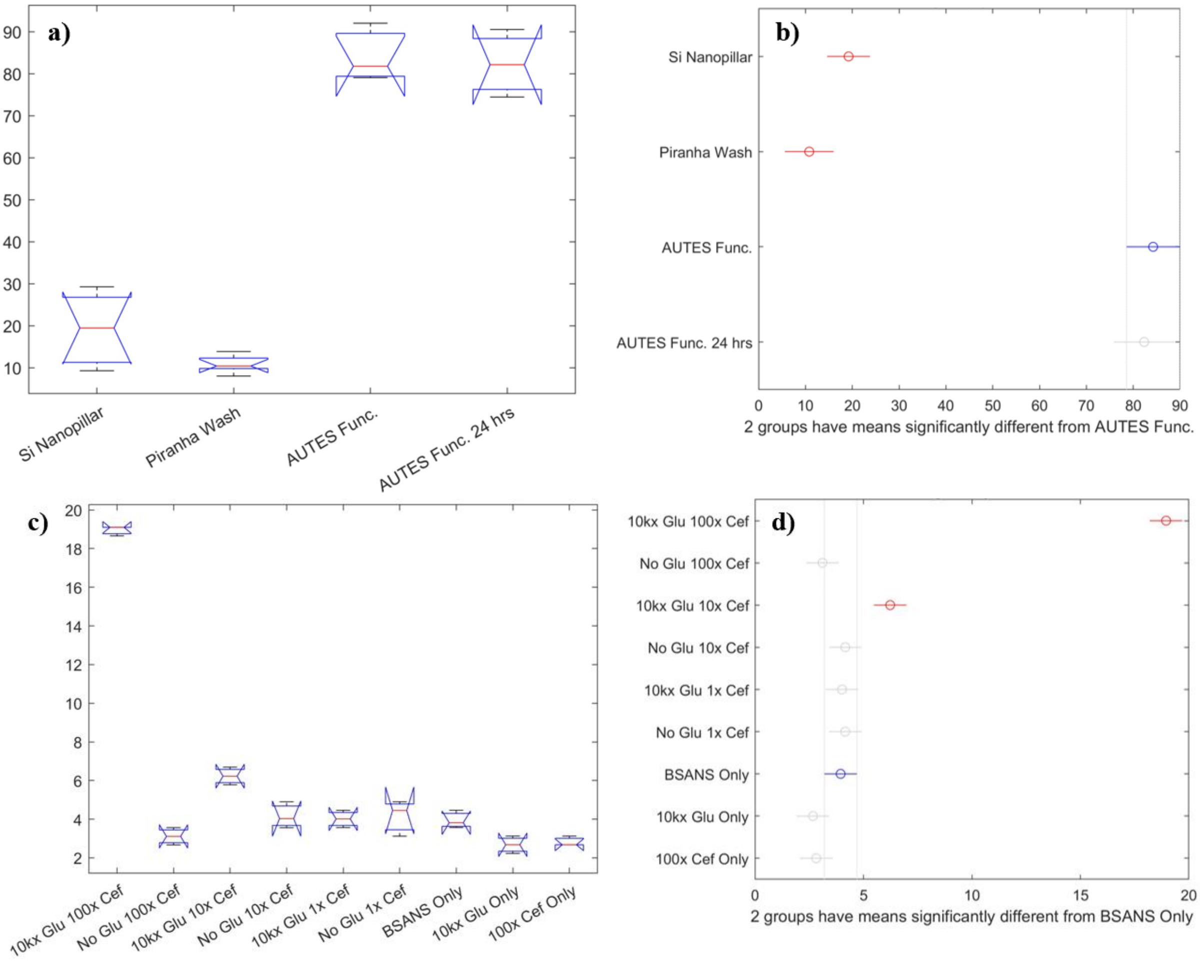

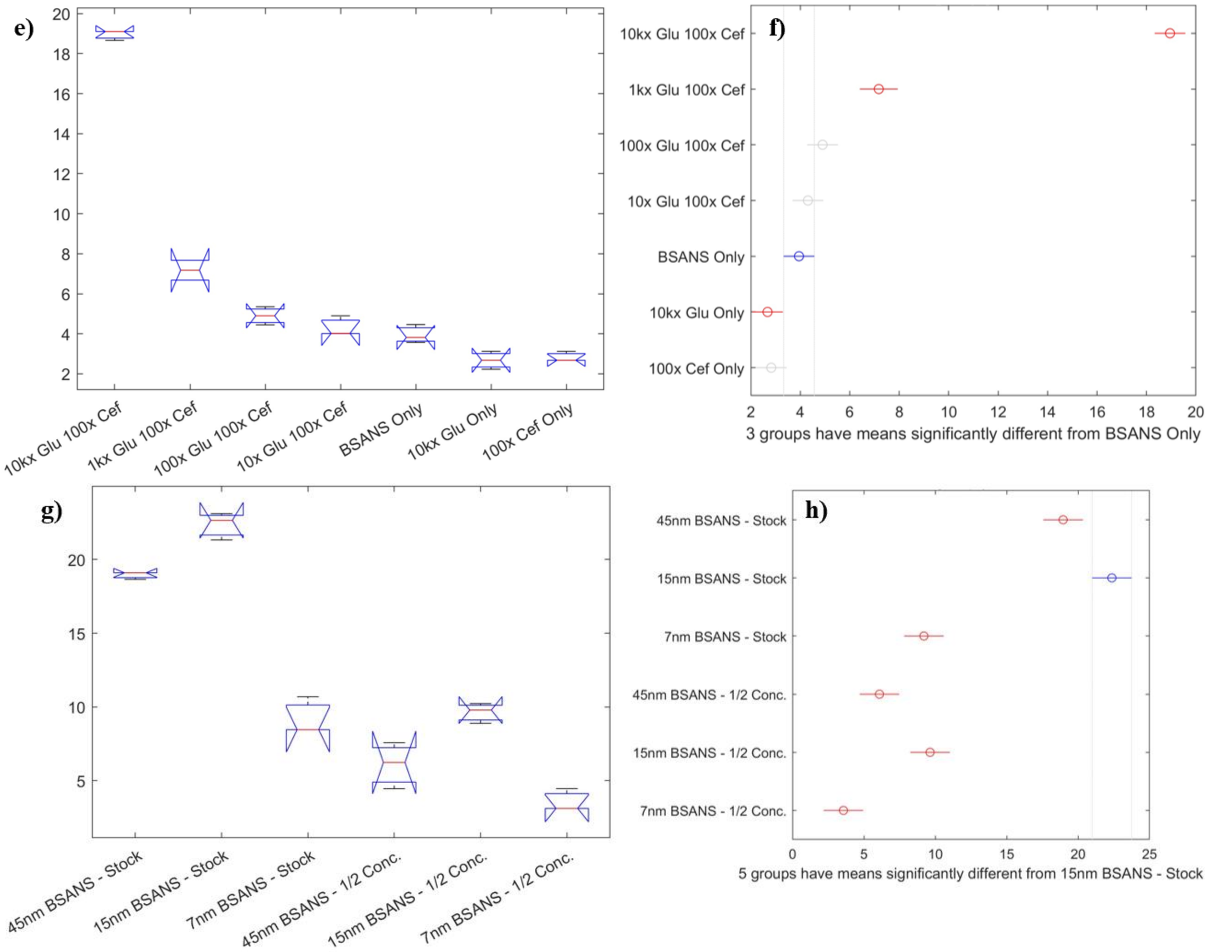

Figure S4. One-way ANOVA analysis for assessing the statistical significance for various experiments. Shown in both box plots and line graphs, the effects of (**a, b**) AUTES SAM coating via contact angle measurements, (**c, d**) cephalexin concentration, (**e, f**) glutaraldehyde concentration, as well as the results from the (**g, h**) varying gold nanoparticle diameter study. For each test the sample size (n) is equal to 3 for each data point, and a P value less than 0.05 was used to measure statistical difference between experiments.

Table S1. One-way ANOVA analysis tables for experiments on (**a**) AUTES contact angle, (**b**) cephalexin concentration, (**c**) glutaraldehyde concentration, and (**d**) gold nanoparticle diameter.

a) ANOVA Table

Source	SS	df	MS	F	Prob>F
Groups	25427	3	8475.65	206.11	1.14447e-14
Error	781.3	19	41.12		
Total	26208.3	22			

b) ANOVA Table

Source	SS	df	MS	F	Prob>F
Columns	631.53	8	78.9412	287.68	2.19902e-17
Error	4.939	18	0.2744		
Total	636.469	26			

c) ANOVA Table

Source	SS	df	MS	F	Prob>F
Groups	591.703	6	98.6171	511.18	1.16213e-14
Error	2.508	13	0.1929		
Total	594.211	19			

d) ANOVA Table

Source	SS	df	MS	F	Prob>F
Columns	823.412	5	164.682	162.21	1.38206e-10
Error	12.183	12	1.015		
Total	835.595	17			
